# The BAriatic surgery SUbstitution and nutrition (BASUN) population: a data-driven exploration of predictors for obesity

**DOI:** 10.1186/s12902-021-00849-9

**Published:** 2021-09-10

**Authors:** Gudrún Höskuldsdóttir, My Engström, Araz Rawshani, Ville Wallenius, Frida Lenér, Lars Fändriks, Karin Mossberg, Björn Eliasson

**Affiliations:** 1 Institute of Medicine, Sahlgrenska Academy, University of Gothenburg, Gothenburg, Sweden; 2 Department of Medicine, Sahlgrenska University Hospital, 413 45 Gothenburg, Sweden; 3 Institute of Health and Care Sciences, Sahlgrenska Academy, University of Gothenburg, Gothenburg, Sweden; 4 Department of Surgery, Region Västra Götaland, Sahlgrenska University Hospital, Gothenburg, Sweden; 5 Institute of Clinical Sciences, University of Gothenburg, Gothenburg, Sweden; 6 Department of Surgery, Sahlgrenska University Hospital, Gothenburg, Sweden; 7 Department of Public Health and Community Medicine, Sahlgrenska University Hospital, Gothenburg, Sweden

**Keywords:** Obesity, Bariatric surgery, Diet, Prospective study, Cohort study

## Abstract

**Background:**

The development of obesity is most likely due to a combination of biological and environmental factors some of which might still be unidentified. We used a machine learning technique to examine the relative importance of more than 100 clinical variables as predictors for BMI.

**Methods:**

BASUN is a prospective non-randomized cohort study of 971 individuals that received medical or surgical treatment (treatment choice was based on patient’s preferences and clinical criteria, not randomization) for obesity in the Västra Götaland county in Sweden between 2015 and 2017 with planned follow-up for 10 years. This study includes demographic data, BMI, blood tests, and questionnaires before obesity treatment that cover three main areas: gastrointestinal symptoms and eating habits, physical activity and quality of life, and psychological health. We used random forest, with conditional variable importance, to study the relative importance of roughly 100 predictors of BMI, covering 15 domains. We quantified the predictive value of each individual predictor, as well as each domain.

**Results:**

The participants received medical (*n* = 382) or surgical treatment for obesity (Roux-en-Y gastric bypass, *n* = 388; sleeve gastrectomy, *n* = 201). There were minor differences between these groups before treatment with regard to anthropometrics, laboratory measures and results from questionnaires. The 10 individual variables with the strongest predictive value, in order of decreasing strength, were country of birth, marital status, sex, calcium levels, age, levels of TSH and HbA1c, AUDIT score, BE tendencies according to QEWPR, and TG levels. The strongest domains predicting BMI were: Socioeconomic status, Demographics, Biomarkers (notably TSH), Lifestyle/habits, Biomarkers for cardiovascular disease and diabetes, and Potential anxiety and depression.

**Conclusions:**

Lifestyle, habits, age, sex and socioeconomic status are some of the strongest predictors for BMI levels. Potential anxiety and / or depression and other characteristics captured using questionnaires have strong predictive value. These results confirm previously suggested associations and advocate prospective studies to examine the value of better characterization of patients eligible for obesity treatment, and consequently to evaluate the treatment effects in groups of patients.

**Trial registration:**

March 03, 2015; NCT03152617.

**Supplementary Information:**

The online version contains supplementary material available at 10.1186/s12902-021-00849-9.

## Background

Obesity is a complex but treatable disease with major individual and societal consequences. Although the World Health Organization (WHO) has emphasized the role of society as well as the individual in preventing obesity, the global prevalence close to tripled between 1975 and 2016 [1]. The Global Burden of Disease Study has recently established that obesity is indeed a major global health challenge, demanding population-wide but country-specific initiatives to mitigate the burden of a wide range of diseases [2].

As formulated by WHO, the fundamental cause of obesity and overweight is an energy imbalance between calories consumed and calories expended against a background environmental and societal factors [1]. There are also links to numerous medical and socioeconomic conditions, e.g., psychiatric, endocrine and cardiovascular disorders [3, 4], which in some cases can presumably be mediated via behavioral, inflammatory and vascular pathways [5]. It is thus quite possible that different mosaics of biological and environmental factors in different individuals, contribute to the development of the disease.

Artificial intelligence in the form of machine learning is increasingly used to discern single factors, or combinations of factors, of importance for defining disease or predicting outcomes. Machine learning techniques are well suited to handle large amounts of data, including variables not commonly used to assess risk in clinical practice, and to identify the smallest number of variables needed for accurate prediction. The use of this method has, e.g., been used to explain variations in obesity prevalence between counties, based on demographic, socioeconomic, health care, and environmental factors [6].

The BASUN study is an ongoing prospective cohort study that follows close to 1000 individuals accepted for treatment of obesity, medical or surgical, in clinical practice in Region Västra Götaland, Sweden for 10 years [7]. An important aim of BASUN is to compare effects and complications of surgical and medical treatment of obesity but the overall goal is to improve the care of individuals with obesity as well as reduce adverse outcomes of treatment. In this study, we applied machine learning algorithms on the extensive clinical information (most of which is not collected in studies in the obesity domain) available for the participants of BASUN. The specific aim of this analysis was to seek out factors strongly linked to severe obesity. In turn such factors can be hypothesis-generating, and can be addressed during the follow-up of BASUN, as well as in prospective trials and clinical practice.

## Methods

### Study design and participants

The design of the BASUN study and the recruited patient cohort have been recently described [7]. To summarize, it is a prospective non-randomized cohort study that originally included 1127 individuals with BMI 35 kg/m^2^ or higher referred for treatment of obesity in clinical practice in Region Västra Götaland, Sweden, between May 2015 and November 2017. Patients were offered medical or surgical treatment of obesity, based on their wishes and whether they met the usual criteria for the different treatment options. The treatment options have been described in detail [7] and included medical treatment with very low energy diet (VLED) for 12–20 weeks followed by an energy restricted diet up to 12 months or surgical treatment (Roux-en-Y gastric bypass (RYGB) or sleeve Gastrectomy (SG)). Apart from regular clinical visits, follow-up according to the study protocol is planned at 2, 5 and 10 years. The Ethical Regional Board of Gothenburg, approved the protocol (application 673–14). Informed consent to participate was obtained from all study participants.

### Anthropometric and laboratory measurements

Demographic data, measurements on height and weight as well as blood tests were collected before the start of treatment. Some blood samples that were important for treatment decisions were analysed directly, while other samples were stored in a biobank [7].

### Questionnaires

We used questionnaires to cover three main areas: gastrointestinal symptoms and eating habits, physical activity and quality of life, and psychological health (previously summarized [7]). The questionnaires included to investigate eating habits were the 21-item Three Factor Eating Questionnaire (TFEQ-R21) [8] and the Questionnaire on Eating and Weight Patterns-Revised (QEWP-R) [9]. To gather information on physical activity and quality of life, the Saltin Grimby (SGQ) physical activity level questionnaire [10], the RAND-36 questionnaire [11], and EuroQol five-dimensional questionnaire (EQ-5D) [12] were included. Two questionnaires were included to investigate psychological health, the Becks Anxiety Inventory (BAI) [13], for the measurement of severity of anxiety and the Patient Health Questionnaire-9 (PHQ-9) [14], a self-reported measure of depression. The Alcohol Use Disorders Identification Test (AUDIT) [15], was used to identify individuals with harmful patterns of alcohol consumption.

### Statistical analysis

Data analysis were performed using R (R Foundation for Statistical Computing, version 4.0.3). Continuous variables are reported as mean (SD) and categorical variables as numbers (n) and proportions (%). Standardized mean difference (SMD) were used to compare group characteristics; SMD is the difference between sample means divided by their pooled standard deviation. A SMD of less than 0.1 was considered non-significant.

The machine learning algorithm random forest has become more common in medical research [16] and was used here to examine the importance of over 100 clinical variables as predictors for body mass index (BMI). Because of the large number of input variables, using fully parameterized regression models would be difficult because of the risk of overfitting. Using random forests with a large number of trees has been shown to be effective in prediction without overfitting.

The variables were also divided manually into 15 clinically similar domains (Socioeconomic status, Age/Sex, Lifestyle and habits, Metabolic disease, Cardiovascular disease, Potential anxiety/depression, Biomarkers for cardiovascular disease and diabetes, Other biomarkers, Medication for cardiovascular disease or diabetes, Psychiatric disease, Gastrointestinal disease, Endocrine conditions, Musculoskeletal disease, Previous surgery and Other conditions). The predictive values of the different domains were assessed as well as the predictive value of each individual variable. Three thousand trees were used for each binary classification model.

The variable importance (described by van der Laan [17]) was computed using a conditional permutation scheme which minimizes the effect of correlation between variables and reliably reflects the impact of each variable [18]. Using random forest variable importance measures include not only the impact each variable individually but also in multivariate interactions with other included variables. Each variable is permuted (removed) randomly and the effect of this permutation on the prediction accuracy is assessed. The variable importance is thus estimated using the difference of accuracy before and after the variable was permuted. Permuting the variable removes the association between that particular variable and the outcome and for important variables, the accuracy of the model decreases. The value of the importance is arbitrary and has been derived from quantifying the change in accuracy by permutation. The permutation accuracy importance used in this manuscript was developed by Strobl and colleagues [18]. A fully adjusted random forest model was also used to analyse and visualize the relationship between the 10 strongest predictors and BMI. These models included 1500 trees. Missing data was handled using multiple imputation by chained equations (MICE) algorithm (mice package in R). Supplementary Figure 1 shows a graphic description of the results before and after imputation.

## Results

The final study population eligible for follow-up in the study and included in this analysis consisted of 1127 individuals of which 971 subsequently started treatment (medical treatment (*n* = 382), RYGB (*n* = 388) and SG (*n* = 201); Table 1). After inclusion, 156 individuals chose not to continue to treatment but are included in the analyses. There were more women in all treatment groups. There were minor differences in mean BMI and age (SMD > 0.1). The majority of the study population was born in Sweden. Using a non-adjusted model, the differences in BMI levels between the sexes and between individuals born in Sweden and outside of Sweden were more pronounced.

**Table 1:** Characteristics of the BASUN population at baseline

	Medical.treatment	RYGB	SG	Discontinued	SMD
**n**	382	388	201	156	
**Male - n (%)**	103 ( 27.2)	87 ( 22.4)	49 ( 24.4)	53 ( 34.0)	
**BMI, kg/m2 - mean (SD)**	41.0 (5.4)	42.5 (4.1)	42.8 (4.9)	41.2 (5.6)	0.223
**Age, years - mean (SD)**	47.6 (14.2)	42.03 (11.3)	40.89 (11.0)	30.5 (159.0)	0.235
**Born in Sweden - n(%)**	226 ( 80.7)	249 ( 87.7)	137 ( 86.2)	50 ( 74.6)	0.194
**Marital status - n (%)**					0.277
Married	145 ( 43.7)	144 ( 40.8)	83 ( 44.6)	37 ( 48.1)	
Cohabitation	61 ( 18.4)	97 ( 27.5)	38 ( 20.4)	8 ( 10.4)	
Relationship w/o cohabitation	9 ( 2.7)	13 ( 3.7)	11 ( 5.9)	3 ( 3.9)	
Single	115 ( 34.6)	98 ( 27.8)	53 ( 28.5)	28 ( 36.4)	
Living w parents	2 ( 0.6)	1 ( 0.3)	1 ( 0.5)	1 ( 1.3)	
**Education - n (%)**					0.268
Elementary school	51 ( 15.5)	42 ( 12.2)	15 ( 8.2)	10 ( 12.8)	
Vocational secondary education	34 ( 10.3)	35 ( 10.2)	24 ( 13.2)	11 ( 14.1)	
Two year secondary education	36 ( 10.9)	57 ( 16.6)	27 ( 14.8)	13 ( 16.7)	
Three year secondary education	75 ( 22.8)	108 ( 31.5)	47 ( 25.8)	19 ( 24.4)	
Started tertiary education	54 ( 16.4)	46 ( 13.4)	40 ( 22.0)	12 ( 15.4)	
University degree	79 ( 24.0)	55 ( 16.0)	29 ( 15.9)	13 ( 16.7)	
**Nicotine - n (%)**					0.232
Non-smoker	162 ( 57.9)	161 ( 49.2)	97 ( 54.2)	34 ( 43.0)	
Ex-smoker	85 ( 30.4)	131 ( 40.1)	61 ( 34.1)	30 ( 38.0)	
Smoker	24 ( 8.6)	24 ( 7.3)	14 ( 7.8)	11 ( 13.9)	
**Hemoglobin, g/L - mean (SD)**	141.5 (12.5)	141.4 (11.0)	141.5 (12.1)	143.2 (12.3)	0.075
**Glucose, mmol/L - mean (SD)**	6.6 (2.3)	6.5 (2.3)	6.4 (1.7)	6.9 (2.4)	0.112
**HbA1c, mmol/mol - mean (SD)**	39.8 (12.1)	39.7 (11.6)	38.8 (9.0)	41.8 (13.3)	0.129
**TSH, mIE/L - mean (SD)**	2.7 (5.5)	2.4 (1.5)	2.7 (2.1)	3.3 (8.5)	0.094
**T4, pmol/L - mean (SD)**	37.4 (389.2)	15.4 (2.2)	15.4 (3.4)	15.5 (2.4)	0.046
**ASAT, μkat/L - mean (SD)**	0.5 (0.2)	0.5 (0.5)	0.5 (0.2)	0.5 (0.2)	0.088
**ALAT, μkat/L - mean (SD)**	0.6 (0.3)	0.7 (0.4)	0.6 (0.5)	0.6 (0.4)	0.084
**Triglycerides, mmol/L - mean (SD)**	1.6 (0.8)	1.6 (1.0)	1.2 (0.4)	1.7 (0.9)	0.354
**HDL, mmol/L - mean (SD)**	1.3 (0.4)	1.3 (0.3)	1.3 (0.2)	1.2 (0.4)	0.120
**LDL, mmol/L - mean (SD)**	3.2 (0.9)	3.3 (0.9)	3.5 (0.9)	3.3 (1.0)	0.170
**Creatinin, mmol/L - mean (SD)**	76.4 (54.1)	71.4 (11.1)	68.2 (9.5)	73.3 (15.8)	0.209
**U-Albumin, mg/L - mean (SD)**	54.4 (262.5)	21.5 (38.5)	13.6 (13.8)	38.4 (98.8)	0.221
**TFEQ CR score - mean (SD)**	2.3 (0.7)	2.3 (0.6)	2.3 (0.7)	2.3 (0.7)	0.064
**TFEQ UE score - mean (SD)**	2.3 (0.8)	2.2 (0.8)	2.3 (0.8)	2.2 (0.8)	0.059
**TFEQ EE score - mean (SD)**	2.49 (0.96)	2.32 (0.93)	2.38 (0.89)	2.3 (1.0)	0.093
**QEWP BE - n (%)**	25 ( 7.8)	20 ( 5.7)	14 ( 7.5)	4 ( 5.9)	0.052
**QEWP BN - n (%)**	0 (0.0)	0 (0.0)	0 (0.0)	0 (0.0)	<0.001
**QEWP BN w CB - n (%)**	0 ( 0.0)	5 ( 1.4)	0 ( 0.0)	0 ( 0.0)	0.085
**AUDIT score - mean (SD)**	2.8 (2.9)	2.8 (2.6)	3.1 (3.0)	2.6 (3.0)	0.078
**BAI, potential anxiety - n (%)**	97 ( 27.6)	74 ( 20.6)	38 ( 20.2)	27 ( 30.7)	0.149
**BAI score - mean (SD)**	11.7 (10.4)	9.5 (8.9)	9.5 (8.7)	12.7 (13.5)	0.187
**EQ5D index value - mean (SD)**	0.8 (0.2)	0.8 (0.2)	0.8 (0.2)	0.8 (0.2)	
**PHQO depression - n (%)**					0.248
No depression	230 ( 66.1)	275 ( 77.0)	144 ( 76.6)	62 ( 70.5)	
Mild depression	67 ( 19.3)	51 ( 14.3)	23 ( 12.2)	9 ( 10.2)	
Moderate depression	33 ( 9.5)	25 ( 7.0)	17 ( 9.0)	13 ( 14.8)	
Serious depression	18 ( 5.2)	6 ( 1.7)	4 ( 2.1)	4 ( 4.5)	
**PHQO score - mean (SD)**	7.6 (6.0)	6.3 (5.1)	6.6 (5.5)	6.9 (6.5)	0.119
**SGQ - n(%)**					0.150
Physically inactive	163 ( 47.1)	155 ( 43.5)	73 ( 39.9)	40 ( 46.0)	
Some light physical activity	156 ( 45.1)	178 ( 50.0)	96 ( 52.5)	40 ( 46.0)	
Regular physical activity and training	22 ( 6.4)	22 ( 6.2)	13 ( 7.1)	7 ( 8.0)	
Regular hard physical training for competition sports	5 ( 1.4)	1 ( 0.3)	1 ( 0.5)	0 ( 0.0)	
**Antihyperglycemics - n (%)**	55 ( 14.4)	56 ( 14.4)	28 ( 13.9)	0 ( 0.0)	0.293
**Antihypertensives - n (%)**	141 ( 36.9)	115 ( 29.6)	59 ( 29.4)	0 ( 0.0)	0.539
**Lipid lowering - n (%)**	51 ( 13.4)	50 ( 12.9)	26 ( 12.9)	0 ( 0.0)	0.279
**Drugs for anxiety/depression - n (%)**	92 ( 24.1)	71 ( 18.3)	53 ( 26.4)	0 ( 0.0)	0.450
**Antipsychotics - n (%)**	13 ( 3.4)	3 ( 0.8)	6 ( 3.0)	0 ( 0.0)	0.168
**Analgetics - n (%)**	83 ( 21.7)	58 ( 14.9)	37 ( 18.4)	0 ( 0.0)	0.394
**Thyroid hormone replacement - n (%)**	50 ( 13.1)	41 ( 10.6)	26 ( 12.9)	0 ( 0.0)	0.289
**ADHD drugs - n (%)**	8 ( 2.1)	2 ( 0.5)	2 ( 1.0)	0 ( 0.0)	0.122
**PPI - n (%)**	51 ( 13.4)	39 ( 10.1)	30 ( 14.9)	0 ( 0.0)	0.319
**Anticoagulants - n (%)**	25 ( 6.5)	18 ( 4.6)	12 ( 6.0)	0 ( 0.0)	0.201
**Inhalations - n (%)**	32 ( 8.4)	35 ( 9.0)	19 ( 9.5)	0 ( 0.0)	0.234
**Other psychiatric disease - n (%)**	25 ( 6.5)	13 ( 3.4)	13 ( 6.5)	6 ( 3.8)	0.094
**Depression/anxiety - n (%)**	42 ( 11.0)	19 ( 4.9)	20 ( 10.0)	7 ( 4.5)	0.155
**Pulmonary disease - n (%)**	43 ( 11.3)	33 ( 8.5)	17 ( 8.5)	4 ( 2.6)	0.176
**Hormonal disorders - n (%)**	36 ( 9.4)	29 ( 7.5)	25 ( 12.4)	9 ( 5.8)	0.129
**Previous surgery - n (%)**	14 ( 3.7)	6 ( 1.5)	5 ( 2.5)	0 ( 0.0)	0.158
**Other medical condition - n (%)**	68 ( 17.8)	54 ( 13.9)	38 ( 18.9)	9 ( 5.8)	0.222
**Other intestinal disease - n (%)**	22 ( 5.8)	6 ( 1.5)	14 ( 7.0)	1 ( 0.6)	0.210
**Diabetes - n (%)**	49 ( 12.8)	55 ( 14.2)	26 ( 12.9)	23 ( 14.7)	0.034
**Joint/back disorders - n (%)**	75 ( 19.6)	61 ( 15.7)	38 ( 18.9)	13 ( 8.3)	0.179
**Sleep apnea - n (%)**	18 ( 4.7)	14 ( 3.6)	12 ( 6.0)	3 ( 1.9)	0.115
**Hypertension - n (%)**	90 ( 23.6)	77 ( 19.8)	41 ( 20.4)	19 ( 12.2)	0.152
**Disease of stomach/gallbladder - n (%)**	23 ( 6.0)	8 ( 2.1)	8 ( 4.0)	3 ( 1.9)	0.125
**Neurological disease - n (%)**	3 ( 0.8)	4 ( 1.0)	3 ( 1.5)	3 ( 1.9)	0.057
**Rheumatological disease - n (%)**	27 ( 7.1)	27 ( 7.0)	15 ( 7.5)	6 ( 3.8)	0.079
**Vitamin/mineral deficiency - n (%)**	7 ( 1.8)	4 ( 1.0)	2 ( 1.0)	1 ( 0.6)	0.055
**Malignancies - n (%)**	7 ( 1.8)	2 ( 0.5)	2 ( 1.0)	1 ( 0.6)	0.069
**IBD/celiac disease - n (%)**	8 ( 2.1)	2 ( 0.5)	3 ( 1.5)	2 ( 1.3)	0.074
**Hyperlipidemia - n (%)**	14 ( 3.7)	18 ( 4.6)	12 ( 6.0)	2 ( 1.3)	0.137
**Cardiovascular disease - n (%)**	6 ( 1.6)	3 ( 0.8)	1 ( 0.5)	0 ( 0.0)	0.103
**Other cardiac disease - n (%)**	21 ( 5.5)	11 ( 2.8)	3 ( 1.5)	3 ( 1.9)	0.121
**Chronic pain - n (%)**	6 ( 1.6)	4 ( 1.0)	6 ( 3.0)	1 ( 0.6)	0.098
**Renal failure - n (%)**	3 ( 0.8)	1 ( 0.3)	1 ( 0.5)	1 ( 0.6)	0.040
**VTE/hypercoagulability - n (%)**	3 ( 0.8)	1 ( 0.3)	4 ( 2.0)	0 ( 0.0)	0.123

There were differences with regard to marital status and education as well as nicotine usage (SMD > 0.1) With regard to previous diabetes, the groups were similar (SMD < 0.1) but there were slight differences in other reported metabolic disease (hyperlipidemia, hypertension and sleep apnea) (SMD > 0.1) as well as levels of HbA1c, glucose and low-density lipoprotein. Information on previous psychiatric illness was self-reported in questions on known diagnosis and pharmaceutical treatment as well as specific questionnaires. Self-reported previous depression or anxiety and treatment for these disorders differed between the groups as well as the results from the questionnaires focusing on depression (PHQ-9) and anxiety (BAI). There was also a difference in reported usage of antipsychotics between the groups. Factors that might influence the choice of bariatric surgery, such as hemoglobin levels, known deficiencies of vitamins and minerals, eating habits assessed by TFEQ and QEWPR questionnaires, AUDIT scores or previous malignancies were not different between the treatment groups (SMD < 0.1) but there was a difference in known gastrointestinal-, pulmonary- and cardiovascular disease.

**Fig. 1:**
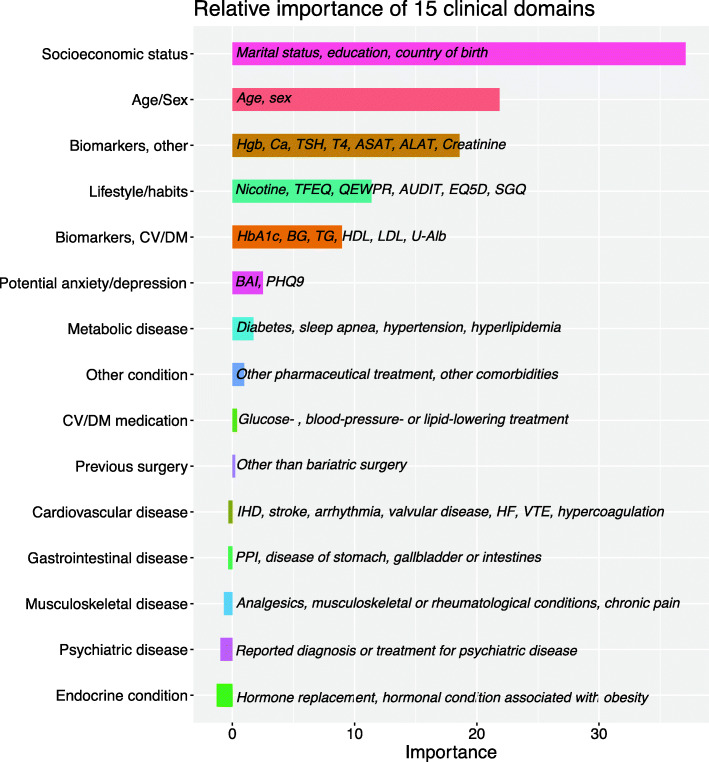
Predictive value of each clinical domain on BMI as computed using a conditional permutation scheme and the variables included within each domain. CV: cardiovascular, DM: diabetes mellitus, Hgb: hemoglobin, Ca: calcium, TSH: thyroid stimulating hormone, T4: thyroxine, ASAT: aspartate aminotransferase, ALAT: alanine aminotransferase, TFEQ: three factor eating questionnaire, QEWPR: Questionnaire on eating and weight patterns, AUDIT: Alcohol use disorders identification test, EQ. 5D: EuroQol five-dimensional questionnaire, SGQ: Saltin Grimby questionnaire, HbA1c: glycated hemoglobin, BG: blood glucose, TG: triglycerides HDL: high density lipoprotein, LDL: low-density lipoprotein, U-Alb: urinary albumin, BAI: Beck anxiety inventory, PHQ-9: Patient health questionnaire-9, IHD: ischemic heart disease, HF: heart failure, VTE: venous thromboembolism, PPI: proton-pump inhibitors, ADHD: attention deficit and hyperactivity disorder

The relative importance of the 15 clinical domains and an overview of the variables included in each domain can be seen in Fig. 1. The distribution of variables within each domain can be seen in more detail in supplementary Table 1. The strongest predictive domains observed were: Socioeconomic status, Age/sex, Other biomarkers (hemoglobin, calcium, TSH, T4, liver transaminases, creatinine), Lifestyle and habits, Biomarkers for cardiovascular disease and diabetes (HbA1c, glucose, TG, HDL, LDL, urinary albumin), Potential anxiety and depression, Metabolic disease, Medication for cardiovascular disease or diabetes and Other conditions. The six remaining domains that had little or no predictive value.

**Fig. 2:**
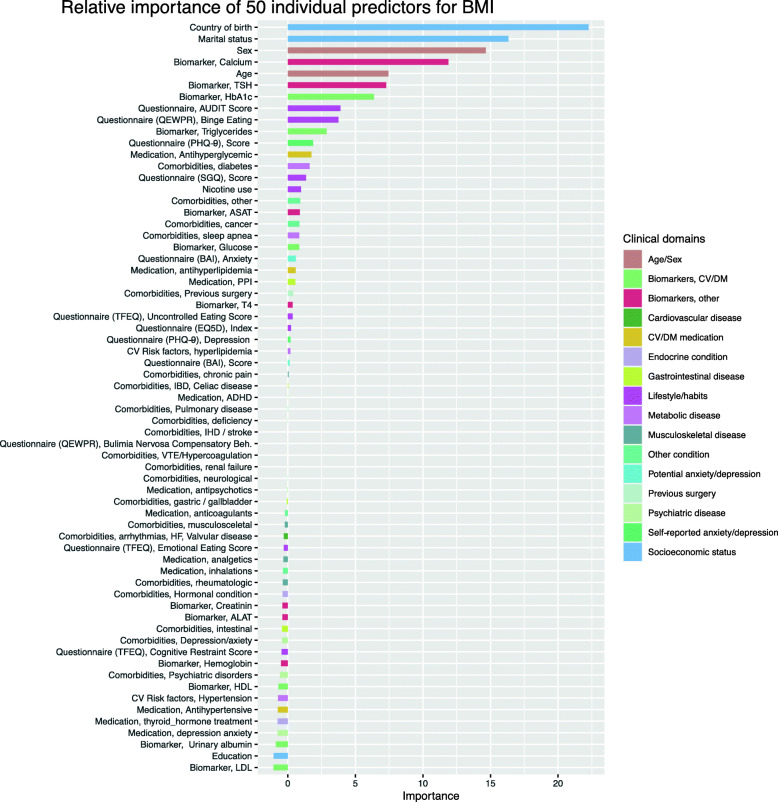
Relative importance of individual predictors on BMI as computed with a conditional permutation scheme. CV: cardiovascular, DM: diabetes mellitus, Hgb: hemoglobin, Ca: calcium, TSH: thyroid stimulating hormone, T4: thyroxine, ASAT: aspartate aminotransferase, ALAT: alanine aminotransferase, TFEQ: Three factor eating questionnaire, QEWPR: Questionnaire on eating and weight patterns, AUDIT: Alcohol use disorders identification test, EQ. 5D: EuroQol five-dimensional questionnaire, SGQ: Saltin Grimby questionnaire, HbA1c: glycated hemoglobin, BG: blood glucose, TG: triglycerides HDL: high density lipoprotein, LDL: low-density lipoprotein, U-Alb: urinary albumin, BAI: Beck anxiety inventory, PHQ-9: Patient health questionnaire-9, IHD: ischemic heart disease, HF: heart failure, VTE: venous thromboembolism, PPI: proton-pump inhibitors, ADHD: attention deficit and hyperactivity disorder

**Fig. 3:**
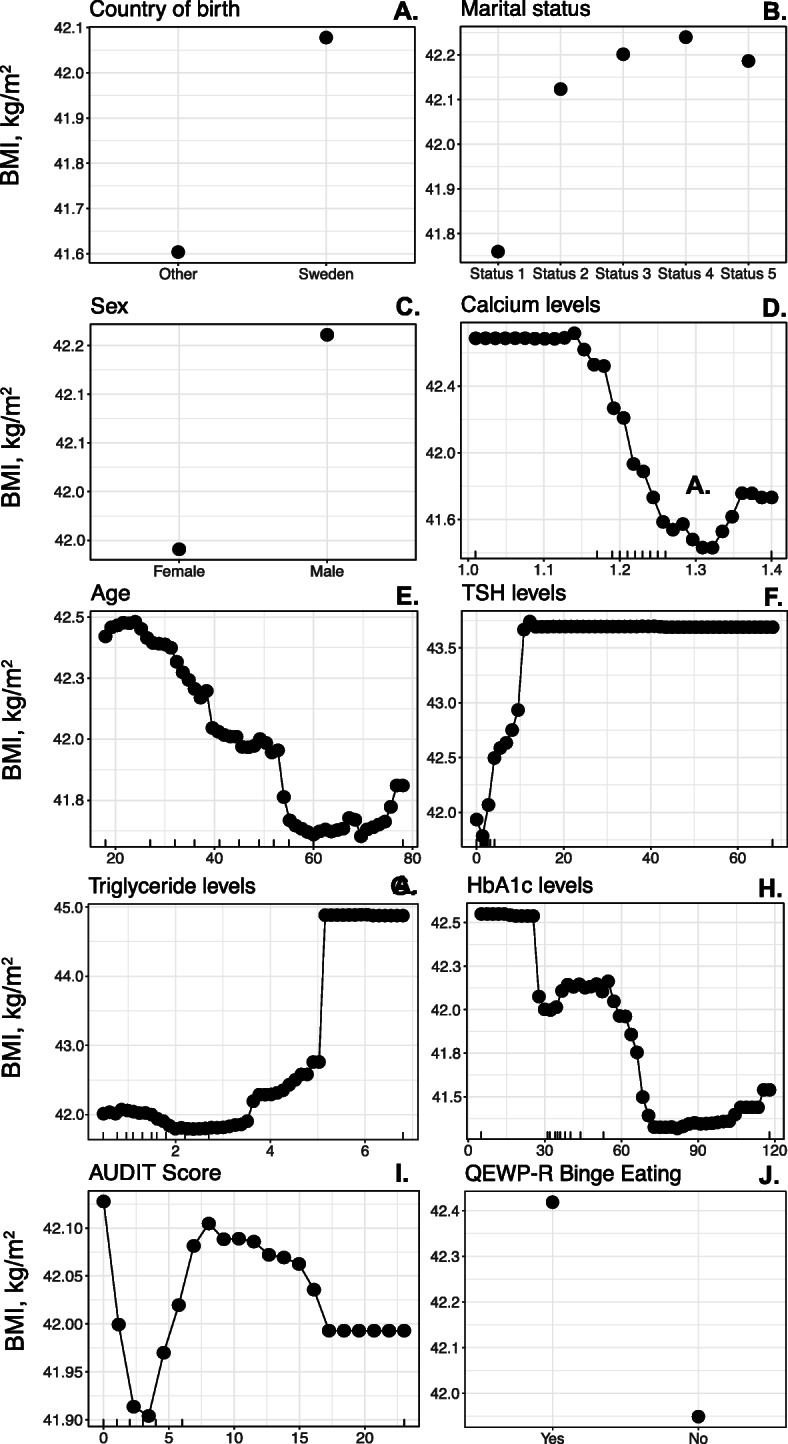
The ten variables with the strongest predictive value for body mass index as analyzed and visualized by random forest. 10% of the population presented by each marking on the x-axis where relevant. Marital status: 1) married, 2) cohabitation, 3) relationship without cohabitation, 4) single, 5) living with parents. TSH: thyroid stimulating hormone, AUDIT: Alcohol use and disorders identification test, QEWP-R: Questionnaire on eating and weight patterns-revised

The 10 individual variables with the strongest predictive value, in order of decreasing strength, were country of birth, marital status, sex, calcium levels, age, levels of TSH and HbA1c, AUDIT scores, binge eating reflected by the QEWPR questionnaire and levels of TG (Fig. 2). The relationship between these 10 variables individually and BMI is graphically presented in Fig. 3. According to the random forest model being born in Sweden, male sex and younger age seem to be associated with higher BMI levels as well as a self-reported tendency of binge eating. Higher levels of triglycerides and thyroid stimulating hormone were also predictors for higher BMI as opposed to lower levels of calcium and HbA1c. For the largest part of the population there was an inverse relationship between AUDIT scores and BMI. Being married (status 1) was associated with lower BMI levels in comparison with living in cohabitation without being married (status 2), a relationship without cohabitation (status 3), being single (status 4) or living with parents (status 5).

## Discussion

In this study of the baseline characteristics of the BASUN population we found that variables associated with socioeconomic status, age, sex, lifestyle and habits are the strongest predictors for BMI levels. Potential depression and anxiety according to questionnaires also have strong predictive values, stronger than self-reported diagnoses or pharmaceutical treatment of these disorders. The predictive values of clinical laboratory measurements such as serum triglycerides and HbA1c, but also to TSH and serum calcium levels were strong. These results confirm previously suggested associations [19–21], but also advocate prospective studies to examine the value of better characterization of patients eligible for obesity treatment, and consequently to evaluate the treatment effects in these groups of patients.

A systematic literature review of machine learning (ML) tools in predicting childhood obesity recently concluded that ML algorithms such as decision trees and artificial neural networks can accurately predict childhood obesity [22]. ML algorithms created to predict obesity in young children mainly focus on height and weight at young age [23], while external factors have been shown to have minor or no influence [24]. Models that focus on predicting obesity in older teenagers have included factors such as eating habits and levels of physical activity [25]. Adult obesity is a complex disease with a multitude of environmental and biological contributing factors. A recent review of various machine learning models used to identify a set of risk factors associated with obesity reported BMI, age, nicotine, blood pressure, blood glucose, lipid profile, adiposity, physical activity, dietary habits and family history as identified risk factors [26]. All of the studies had obesity or overweight as the outcome, not BMI as included in the present study. Models for predicting obesity in adults have to include large magnitude of diverse data. Previous studies have used data from food sales to predict obesity and have shown that the strongest categories in predicting obesity on a country level were baked goods/flours, cheese and carbonated drinks. A reported limitation of this study was that it was unclear if the diet composition was a true cause of obesity or simply a surrogate for sedentary behavior [27]. Predictive decision tree algorithms have also been used to predict metabolic syndrome and to rank behaviors that lead to long-term success after RYGB surgery [28, 29] and data from the Scandinavian Obesity Surgery Registry has been used to compare the capability of different machine learning algorithms in predicting severe complications of surgery. Although the algorithms performed well on the training data, none of the methods included successfully predicted these outcomes when applied to data outside of the training set, indicating the difficulty of applying results from such analyses to real life [30].

The majority of the participants included in our study have similar BMI. Some of the variables presented as having high predictive value, such as liver transaminases, triglycerides and HbA1c levels are more likely to be secondary to obesity. The extreme levels of TSH were only seen in a small percentage of participants with the majority of individuals having levels closer to normal range. The relationship between untreated hypothyroidism and higher BMI levels is not surprising, but the relationship between calcium and BMI was not. Higher BMI is more likely to be associated with lower calcium levels because of relative vitamin D deficiency in individuals with obesity due to d-vitamin sequestration in adipose tissue [31]. Generally, being married has been related to higher BMI levels although this has been shown to differ by gender, age and even ethnicity in studies based on data from the United States [32]. Our results indicate that being married is related to lower BMI levels. The largely Swedish population and lack of variety in ethnicity might in our study explain these differences in comparison to other studies. The value of education as an individual predictor was much lower than many of the other variables although the ‘Socioeconomic status’ domain was the strongest predictive domain. This suggest that it is the actual combination of certain factors that matters.

The high predictive value of answers from questionnaires indicates the importance of these in the evaluation and treatment of individuals with obesity. Scores from the PHQ-9, QEWPR and AUDIT questionnaires were shown to have much higher predictive value than self-reported psychiatric disease or pharmaceutical treatment for anxiety and/or depression. The population included generally reported a low level of physical activity according to the Saltin Grimby questionnaire, with 90–95% of the patients reporting sedentary or low levels of activity only*.* However, this variable was not one of the individual variables with the strongest predictive value. When the effect of levels of TSH and liver transaminases are considered within the ‘Biomarkers, other’ domain, it is likely that the predictive effect of this domain might be misleading as these markers are more likely to be secondary to obesity. This might also be the case with the effect of TG levels within the ‘Biomarkers, CV/DM domain’.

The prevalence of obesity differs between men and women to varying degrees in different parts of the world. The reasons for this are considered to be multifaceted [33]. In the present study, as well as studies on obesity in general, there were more women, but we observed extremely high BMI levels in younger men and the average BMI was higher among the men. A higher mean pre-operative BMI in males has been reported previously [34]. The inverse relationship between age and BMI as well as the fact that most of the individuals included had normal HbA1c levels might explain the observed association of higher BMI levels with lower HbA1c levels as poor glycemic control might not yet have developed in the younger individuals. The fact that the difference in BMI levels between the sexes and depending on country of birth were more pronounced using a non-adjusted model indicate that at least some of the associations of sex and country of birth are mediated by other factors.

A strength of the present study is the use of a large number of diverse validated variables, including socioeconomic information, biomarkers, psychiatric health, eating habits, alcohol, nicotine, levels of physical activity, previous diseases as well as pharmaceutical treatment. The study includes close to 1000 patients and planned long-term follow-up. The choice of treatment is based not only on clinical guidelines but also on the patient’s preferences. This approach reflects the treatment as it is in clinical practice. The BASUN study includes a heterogenous population of individuals with obesity, not only focusing on established comorbidities. The population included can be considered representative and is comparable to participants in other larger obesity treatment studies such as the Swedish Obese Subjects (SOS) ( [35]), as well as the OPTITWIN [36], DIETFITS [37] and POUNDS LOST studies [38]. All of these included a predominantly female population and similar age groups (39–52 years). The BMI levels in the SOS study were also between 41 and 42 kg/m^2^ but slightly lower in the other studies (33–39 kg/m^2^). Our statistical methods, random forest variable importance measure, covers the impact of each predictor as well as multivariable interactions, and using conditional random forest models has the advantage of minimizing the effect of correlations between different variables.

There were also limitations in the study. The individuals included in this study have been referred and accepted for treatment of obesity. The population might therefore differ from the general overweight population seen in society in general as well as in clinical practice in other settings, which can limit the external validity. The differences in certain baseline characteristics were expected due to non-randomization. The study is largely based on self-reported data and there is a risk that individuals that have the most severe psychiatric disorders will not answer the questionnaires or that individuals that do not succeed with their treatment might not report back during the follow-up period. This might introduce bias. There might be a certain economic aspect in the choice of treatment as participants included in the medical treatment group pay for the VLED products themselves. Information on income or employment status was not included, but the VLED diet is not more costly than a normal diet and there were only minor differences between the groups with regards to education and marital status which could reflect economic status to some degree. An inclusion criterion for the study was that participants could understand Swedish which excluded some participants with country of birth other than Sweden, and we have not collected genetic data or the family history of the patients.

An important aim of the analysis in the present study was to seek out variables that could be hypothesis generating and useful in early risk prediction of obesity. Before the results of our study can be applied directly in clinical practice, further studies are needed. However, the factors with the strongest predictive value described in this study, such as scores from questionnaires may be of value when choosing treatment options for obesity, both medical and surgical. Comparing different types of ML methods on the BASUN data and dividing the population by class of obesity might be of value. Prospective studies using ML techniques including individuals that are overweight and not yet obese, might also add valuable information on predictive factors for obesity. Planned analyses of follow-up data from BASUN will be used to find predictive variables for successful obesity treatment.

## Conclusions

Variables associated with lifestyle, habits, age, sex and socioeconomic status are the strongest predictors for BMI levels. Self-reported anxiety and depression through questionnaires also have strong predictive value, stronger than self-reported diagnosis or pharmaceutical treatment of these disorders. We propose that future studies should examine the value of wider characterization of patients treated for obesity.

## Supplementary Information

**Additional file 1: Supplementary figure 1.** Missing data patterns before and after imputation with MICE.

**Additional file 2: Table S1.** Individual variables included in each clinical domain. *Questionnaires.

## Data Availability

Data and material are not available due to the nature of this prospective study. The datasets used and/or analysed during the current study available from the corresponding author on reasonable request. All methods were carried out in accordance with relevant guidelines and regulations.

## Abbreviations

T2D: Type 2 diabetes
BMI: Body mass index
MT: Medical treatment
RYGB: Roux-en-Y gastric bypass
SG: Sleeve gastrectomy
