# Supplementary material for: The BAriatic surgery SUbstitution and nutrition (BASUN) population: a data-driven exploration of predictors for obesity
Source: BMC Endocr Disord. 2021 Sep 10;21:183. doi: 10.1186/s12902-021-00849-9 (PMC8431862; doi:10.1186/s12902-021-00849-9)
Supplement: Supplementary file 1 — Additional file 1: Supplementary figure 1. Missing data patterns before and after imputation with MICE. [file 12902_2021_849_MOESM1_ESM.pdf]

Supplementary figure 1. Missing data patterns before and after imputation with MICE

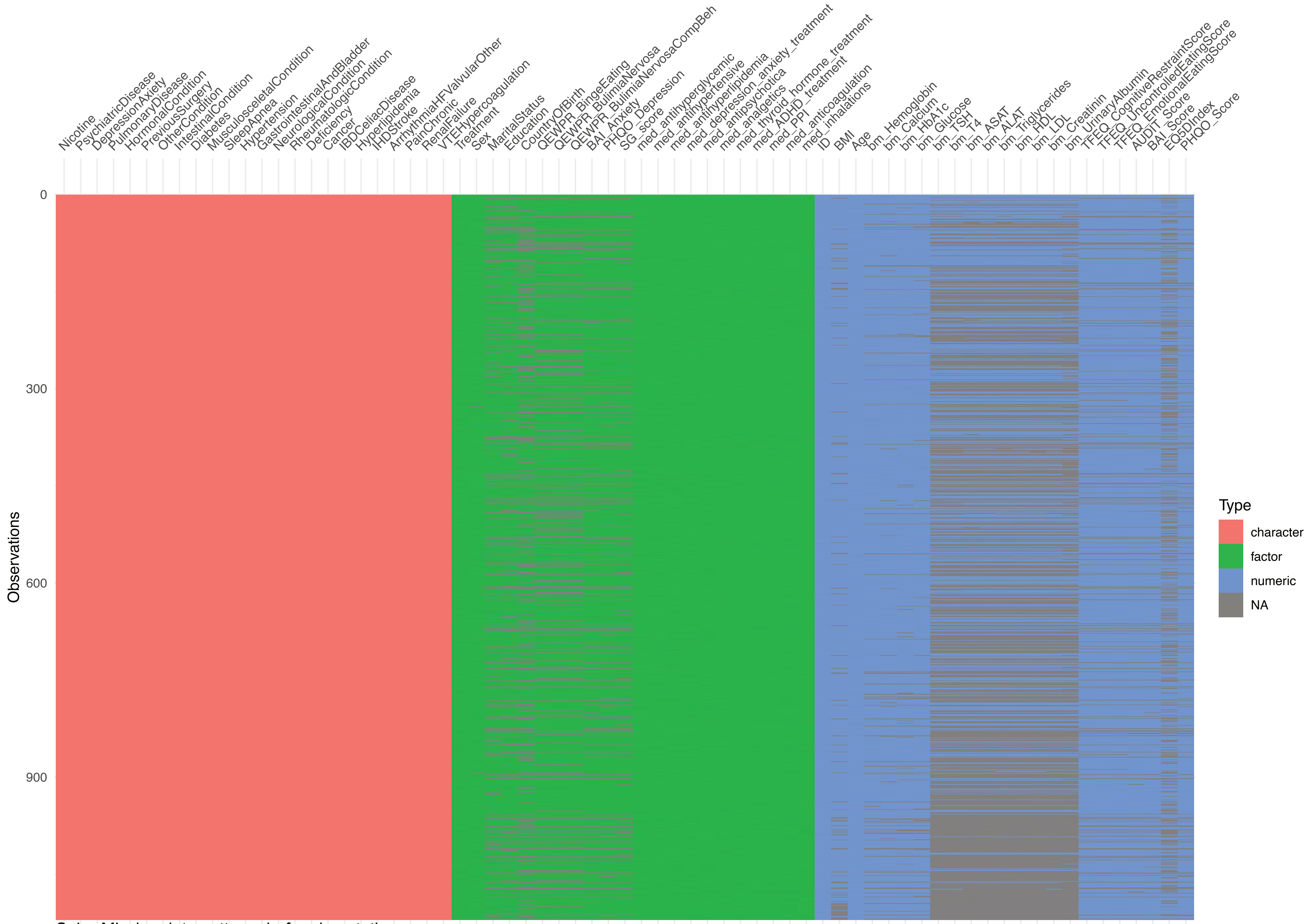


S-1a. Missing data patterns before imputation

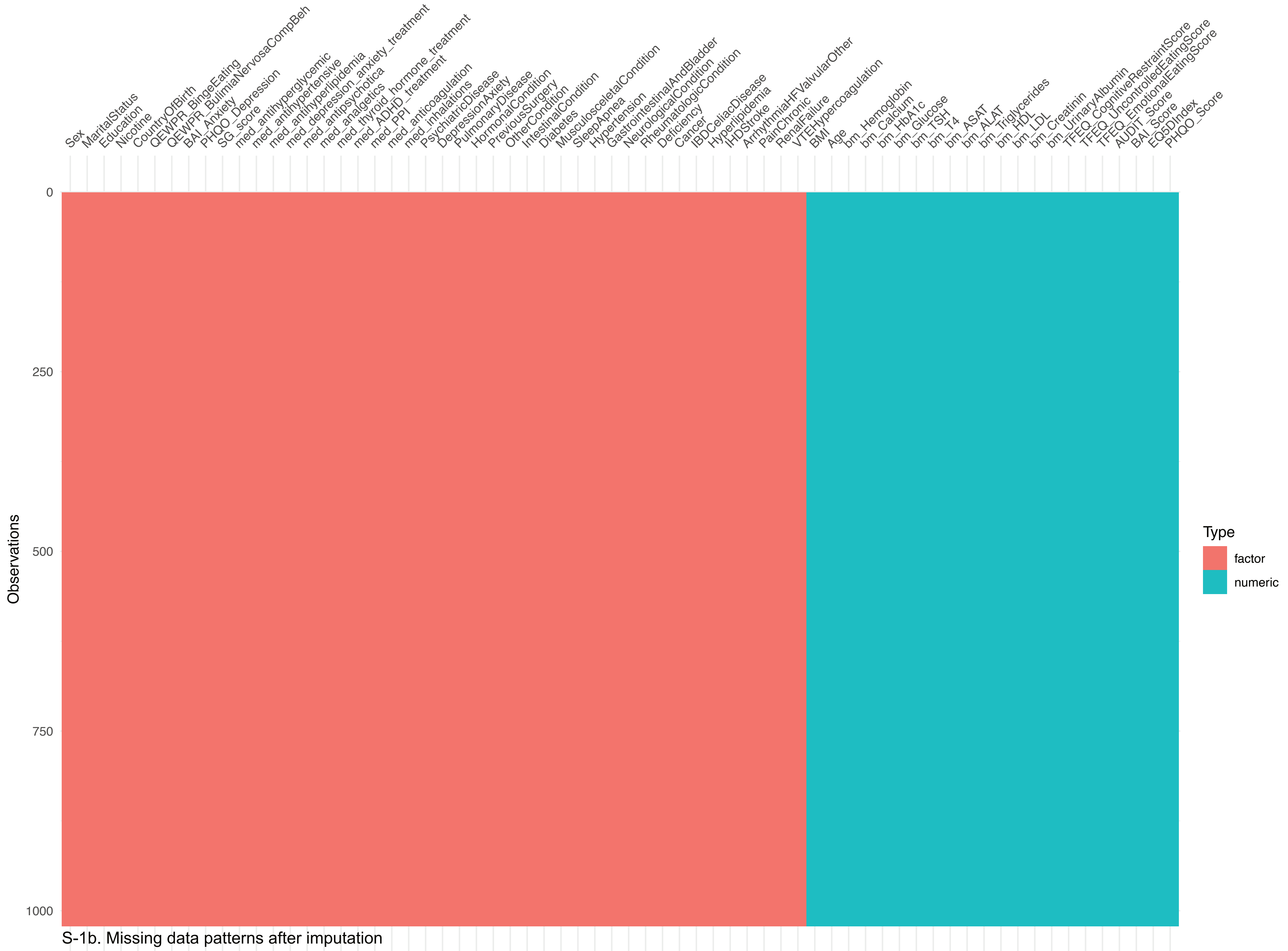


S-1b. Missing data patterns after imputation
