# Supplementary material for: The BAriatic surgery SUbstitution and nutrition (BASUN) population: a data-driven exploration of predictors for obesity
Source: BMC Endocr Disord. 2021 Sep 10;21:183. doi: 10.1186/s12902-021-00849-9 (PMC8431862; doi:10.1186/s12902-021-00849-9)
Supplement: Supplementary file 2 — Additional file 2: Table S1. Individual variables included in each clinical domain. *Questionnaires. [file 12902_2021_849_MOESM2_ESM.docx]

| **Clinical domain** | **Variables included** |
| --- | --- |
| Age / Sex | Age, sex |
| Lifestyle/ habits | Nicotine use  TFEQ*  QEWPR*  AUDIT*  EQ5D index*  SGQ * |
| Socioeconomic status | Marital status  Education  Country of birth |
| Biomarkers, other | Hemoglobin  Calcium  TSH and T4  ASAT and ALAT  Creatinine |
| Biomarkers, CV/DM | HbA1c  Glucose  TG, HDL, LDL  Urinary albumin |
| Potential anxiety/depression | BAI*  PHQ9* |
| CV/DM medication | Glucose-lowering drugs  Blood-pressure lowering drugs  Lipid-lowering drugs |
| Metabolic disease | Diabetes  Sleep apnea  Hypertension  Hyperlipidemia |
| Other condition | Anticoagulants  Inhalations  Pulmonary disease  Neurological disease  Vitamin/mineral deficiency  Previous cancer  Renal failure  Other comorbidities |
| Previous surgery | Previous surgery other than bariatric surgery |
| Cardiovascular disease | Ischemic heart disease  Stroke  Cardiac arrythmia  Cardiac valvular disease  Heart failure  Venous thromboembolic event  Hypercoagulation |
| Gastrointestinal disease | Proton pump inhibitors  Disease of the stomach  Disease of the gallbladder  Disease of the intestines (including inflammatory bowel disease and celiac disease) |
| Musculoskeletal disease | Analgesics  Musculoskeletal condition (including disease of joints, vertebral column and muscles)  Rheumatological disease  Chronic pain |
| Psychiatric disease | Antianxiety agents  Antidepressants  ADHD treatment  Depression  Anxiety  Other psychiatric disease |
| Endocrine conditions | Thyroid hormone replacement agent  Other hormone replacement agent  Hormonal condition associated with obesity (hypothyroidism, polycystic ovarian disease) |

Legend s-Table 1: Individual variables included in each clinical domain. *Questionnaires
